# The Diagnostic Approach towards Combined Hepatocellular-Cholangiocarcinoma—State of the Art and Future Perspectives

**DOI:** 10.3390/cancers15010301

**Published:** 2023-01-01

**Authors:** Johannes Eschrich, Zuzanna Kobus, Dominik Geisel, Sebastian Halskov, Florian Roßner, Christoph Roderburg, Raphael Mohr, Frank Tacke

**Affiliations:** 1 Department of Hepatology and Gastroenterology, Campus Virchow Klinikum and Campus Charité Mitte, Charité Universitätsmedizin Berlin, Augustenburger Platz 1, 13353 Berlin, Germany; 2 Department for Radiology, Campus Virchow Klinikum, Charité Universitätsmedizin Berlin, Augustenburger Platz 1, 13353 Berlin, Germany; 3 Department of Pathology, Charité Universitätsmedizin Berlin, Charitéplatz 1, 10117 Berlin, Germany; 4 Clinic for Gastroenterology, Hepatology and Infectious Diseases, Medical Faculty of Heinrich Heine University Düsseldorf, University Hospital Düsseldorf, Moorenstraße 5, 40225 Düsseldorf, Germany

**Keywords:** combined hepatocellular-cholangiocarcinoma, hepatocellular carcinoma, cholangiocarcinoma, diagnostic approach, biomarker, liquid biopsy, radiomics, artificial intelligence, future outlook

## Abstract

**Simple Summary:**

Combined hepatocellular-cholangiocarcinoma (cHCC-CCA) is a specific form of primary liver cancer with features of both hepatocellular and biliary tract cancer. This review provides an overview about the current state-of-the-art diagnostic workup in patients with cHCC-CCA and discusses future perspectives. Differentiating cHCC-CCA from other liver tumours is crucial for the optimal treatment decision. The diagnostic workup to date mainly consists of the evaluation of the clinical features, laboratory tests, radiological imaging and histopathological evaluation of biopsies. During that process, several potential difficulties arise, which we discuss in this review.

**Abstract:**

Combined hepatocellular-cholangiocarcinoma (cHCC-CCA) is a rare primary liver cancer which displays clinicopathologic features of both hepatocellular (HCC) and cholangiocellular carcinoma (CCA). The similarity to HCC and CCA makes the diagnostic workup particularly challenging. Alpha-fetoprotein (AFP) and carbohydrate antigen 19-9 (CA 19-9) are blood tumour markers related with HCC and CCA, respectively. They can be used as diagnostic markers in cHCC-CCA as well, albeit with low sensitivity. The imaging features of cHCC-CCA overlap with those of HCC and CCA, dependent on the predominant histopathological component. Using the Liver Imaging and Reporting Data System (LI-RADS), as many as half of cHCC-CCAs may be falsely categorised as HCC. This is especially relevant since the diagnosis of HCC may be made without histopathological confirmation in certain cases. Thus, in instances of diagnostic uncertainty (e.g., simultaneous radiological HCC and CCA features, elevation of CA 19-9 and AFP, HCC imaging features and elevated CA 19-9, and vice versa) multiple image-guided core needle biopsies should be performed and analysed by an experienced pathologist. Recent advances in the molecular characterisation of cHCC-CCA, innovative diagnostic approaches (e.g., liquid biopsies) and methods to analyse multiple data points (e.g., clinical, radiological, laboratory, molecular, histopathological features) in an all-encompassing way (e.g., by using artificial intelligence) might help to address some of the existing diagnostic challenges.

## 1. Introduction

Combined hepatocellular-cholangiocarcinoma (cHCC-CCA) is a primary liver cancer (PLC) which exhibits both hepatic and biliary differentiation. This “biphenotypic” tumour is a rare entity (accounts for less than 5% of all PLCs) [1] and is associated with a dismal prognosis. Studies show that cHCC-CCA have either an intermediate prognosis between hepatocellular (HCC) and cholangiocellular carcinoma (CCA) [2,3,4] or lower survival rate than both liver tumours [5,6]. cHCC-CCA has first been described in 1903 by H Gideon Wells [7]. Since then, the definition and terminology related to this malignancy has constantly evolved. In the literature, common nomenclatures which are often used synonymously to cHCC-CCA include “mixed hepatocellular- cholangiocarcinoma”, “mixed hepatobiliary carcinoma”, “hepato-cholangiocarcinoma” and “hybrid HCC-CC”. Interest has increasingly grown because of the ambiguous phenotypic and morphological character of cHCC-CCA, which makes the diagnosis challenging [8]. Further, it has long been a matter of dispute whether cHCC-CCA is a subtype of HCC or CCA or represents a distinct entity [9]. The risk factors, age of onset, sex ratio and initial symptoms are comparable to ordinary HCC and CCA [10]. Despite recent developments in non-invasive methods, radiological imaging and analysis of serum tumour markers are insufficient for a definitive diagnosis. To date, histopathological evaluation of the biopsy or surgical specimen plays the key role in the diagnosis of cHCC-CCA. However, there is a continued progress in discovering molecular alterations which could be utilised as promising biomarkers for cHCC-CCA detection and early diagnosis. Liquid biopsies, radiomics and neuronal networks are further approaches with the potential to improve the diagnostic workup for cHCC-CCA patients in the future.

This review is structured along the diagnostic patient journey. We provide an overview of the present state of knowledge and discuss challenges and opportunities.

## 2. Clinical Presentation

Due to its rareness, studies that aim to describe clinical characteristics of cHCC-CCA are limited by small patient cohorts and low statistical power [11]. Therefore, the clinical presentation of this malignancy is still poorly understood and debatable [12,13]. In general, cHCC-CCA patients present with features characteristic for HCC and CCA. In early stages, patients may remain asymptomatic, but eventually they develop signs such as obstructive jaundice, fatigue, weight reduction, pruritus, ascites, acute cholangitis, fever, palpable gallbladder, or hepatomegaly [14]. Interestingly, a retrospective study by Chantajitr et al. found that abdominal pain was the first symptom observed in 80% of cHCC-CCA patients compared to HCC (56% of patients) [15].

Risk factors for cHCC-CCA differ in various geographic regions or patient populations owing to differences in diet, living conditions and incidence of infectious diseases [11,16]. Taking into account that cHCC-CCA contains areas of both hepatocytic and cholangiocytic differentiation, risk factors for both HCC and CCA are important for pathogenesis of cHCC-CCA [14]. Lee et al. showed that hepatitis B virus (HBV) infection was significantly more frequently found in cHCC-CCA patients and HCC patients than CCA patients [17]. Analogous to HCC, male sex and middle age are associated with higher likelihood for cHCC-CCA development [14,18,19,20]. Further, Shetty et al. reported a higher prevalence of liver cirrhosis in persons with cHCC-CCA or HCC, in contrast to CCA patients [21].

## 3. Laboratory Features

In addition to clinical features, laboratory tests are of relevance in the diagnosis of cHCC-CCA. Alpha-fetoprotein (AFP) and carbohydrate antigen 19-9 (CA 19-9) are established circulating tumour markers related with HCC and CCA, respectively [22,23]. According to studies, they have the potential to be used as a diagnostic clue in cHCC-CCA as well [24]. Li et al. found that increased AFP and CA 19-9 was observed in 62.2% and 22.2% of cHCC-CCA patients, respectively [24]. It is interesting to note that AFP level seems to be lower, whereas the level of CA 19-9 is significantly higher in cHCC-CCA when compared with HCC [15]. Although these tumour markers are not specific and might be elevated in various conditions, cHCC-CCA should seriously be taken into consideration when both markers are increased concomitantly [25]. Further, CA 19-9 or AFP elevation inconsistent with imaging findings (for instance, elevated CA 19-9 and the presence of imaging features characteristic for HCC, or elevated AFP together with CCA imaging findings) should also alert the physician to the possibility of cHCC-CCA [11]. Nevertheless, Li et al. demonstrated that a concomitant elevation of AFP and CA 19-9 was found only in approximately 15% of cHCC-CCA patients, suggesting a limited sensitivity of these blood tests [24]. Thus, there is a need for more accurate non-invasive biomarkers, which is an emerging field of research.

## 4. Radiological Features 

**Figure 1:**
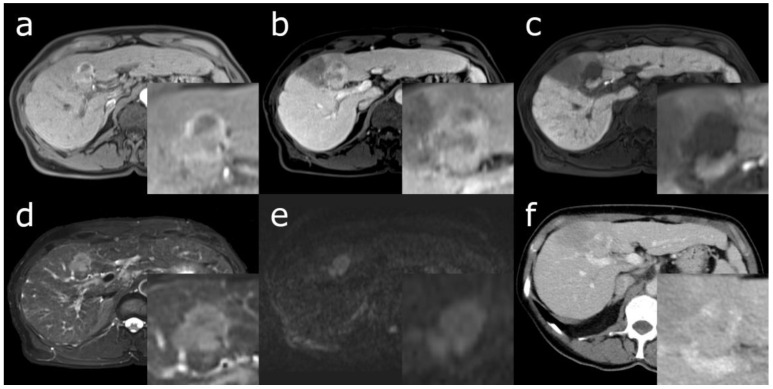
52-year-old female with liver cirrhosis secondary to chronic hepatitis C infection, Child-Pugh grade A. Axial Gd-EOB enhanced MRI of a singular cHCC-CCA in segment IVa with predominant HCC histopathology and macrovascular invasion. (**a**) Rim arterial phase hyperenhancement; (**b**) “washout” in portal venous phase with adjacent parenchymal perfusion deficits; (**c**) homogenous Gd-EOB uptake deficiency in hepatobiliary phase; (**d**) hyperintensity and nodule-in-nodule appearance in a T2-weighted image with fat saturation; (**e**) homogenous diffusion restriction in diffusion weighted imaging (B-value 800); (**f**) CE-CT from the same day showing rim enhancement in the venous phase.

**Figure 2:**
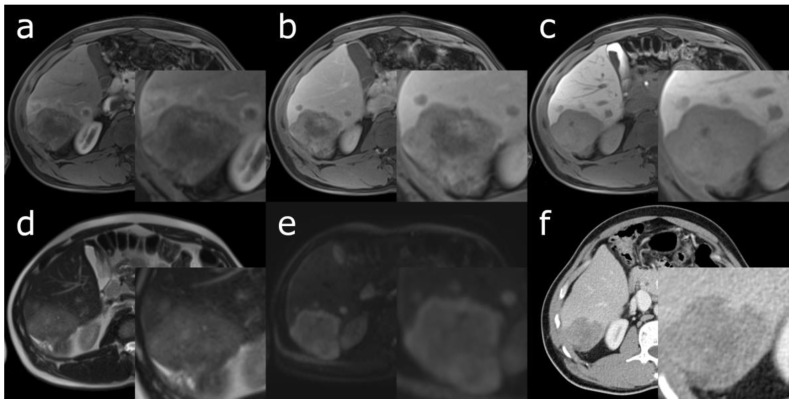
48-year-old male with chronic hepatitis B infection in the absence of cirrhosis. Axial Gd-EOB-enhanced MRI of a cHCC-CCA in segment VI with predominant CCA histopathology, smaller satellite lesions and microvascular invasion. (**a**) Targetoid enhancement of the primary lesion in arterial phase, whereas the satellite lesions show rim hyperenhancement; (**b**) progressive targetoid enhancement in the portal venous and (**c**) hepatobiliary phase; (**d**) hyperintensity in a T2-weighted image; (**e**) targetoid appearance in diffusion weighted imaging (B-value 800); (**f**) CE-CT in venous phase 3 months prior to MRI showing targetoid hypointensity of the primary lesion.

The imaging features of cHCC-CCA overlap with those of HCC and CCA, largely determined by the predominant histopathological component among the two entities [26,27]. cHCC-CCAs with a larger HCC component are more likely to show imaging features compatible with HCC, such as non-rim arterial phase hyperenhancement and portal venous phase “washout” in contrast-enhanced (CE) computed tomography (CT) or magnetic resonance imaging (MRI) (Figure 1) [28,29,30]. Conversely, a larger CCA component is associated with imaging features suggesting CCA, such as targetoid appearance in diffusion weighted imaging or a central area with progressive post-arterial phase enhancement (Figure 2). Due to this overlap, there is a lack of established imaging features that are exclusive to cHCC-CCA [31].

In some cases, a lesion may show two areas with distinct enhancement patterns that suggest HCC or CCA, respectively. Aoki et al., in a retrospective study of CE-CT of 14 cHCC-CCA lesions, reported that 9 lesions (64.3%) consisted of a peripheral zone with early-phase enhancement and late-phase washout, and a central zone that enhanced only in the late phase [32]. This correlated with the pathohistological findings, where HCC-predominant components were found in the periphery, CCA-predominant components with fibrous stroma in the centre, and an intermediate transitional zone. Sanada et al. identified a similar CE-CT enhancement pattern and similar histopathology in 4 out of 9 cHCC-CCA lesions (44.4%) [33]. Gigante et al. went on to find that this imaging pattern had 48% sensitivity and 81% specificity for differentiating cHCC-CCA from CCA [34]. However, in a population with a high proportion of mixed type cHCC-CCAs, where HCC and CCA components intermingle, only 4 in 30 lesions (13.3%) had this finding [35].

A further possibility for improving non-invasive differentiation of cHCC-CCA is to correlate imaging features with the abovementioned tumour markers. In a retrospective study of CEUS and CE-CT in 45 patients with cHCC-CCA, Li et al. found that half of patients had serum biomarkers that were discordant with the diagnosis suggested by imaging [24]. Furthermore, Yang et al. integrated Liver Imaging and Reporting Data System (LI-RADS) categories with CA 19-9 and AFP to achieve high specificity for differentiating cHCC-CCA from HCC or CCA; however, it was at the cost of poor sensitivity, since only 3 out of 35 patients (8.6%) had simultaneously elevated CA 19-9 and AFP [36].

The Liver Imaging and Reporting Data System (LI-RADS) is a widely used standard published by the American College of Radiology (ACR) for CT, MRI and contrast enhanced ultrasonography (CEUS) in liver imaging in a high-risk population for HCC [37]. It defines major and ancillary imaging features that suggest either HCC or non-HCC malignancy. While LI-RADS excels at differentiating HCC from other focal liver lesions, as many as half of cHCC-CCAs may be categorised as ‘LR-5: definitely HCC’, in particular in populations with a higher proportion of liver cirrhosis [38,39,40,41,42]. This is a diagnostic dilemma, as—uniquely among cancers—the diagnosis of HCC may be made without histopathological confirmation if a lesion shows typical imaging morphology for HCC and occurs in a cirrhotic liver [43]. This could lead to misclassification and subsequently suboptimal treatment of cHCC-CCA as HCC, highlighting the need to consider the possibility of non-HCC lesions mimicking HCC [41,44,45]. 

## 5. Role of Biopsy

**Figure 3:**
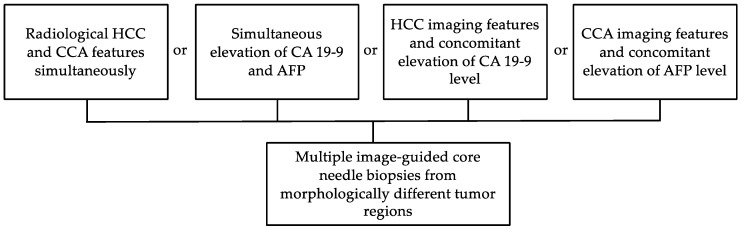
Indications for liver biopsies in the context of cHCC-CCA.

Image-guided core needle liver biopsies are of benefit before starting the appropriate therapy in cHCC-CCA patients. Overall, a biopsy should seriously be considered in the presence of HCC imaging features and concomitant changes in CA 19-9 level, presence of radiological hallmarks of CCA together with high levels of AFP, elevation of these both tumour markers or, finally, the appearance of radiological HCC and CCA features simultaneously (Figure 3). Currently, the diagnosis of cHCC-CCA based on the tumour biopsy alone remains challenging (with a 33% sensitivity and 100% specificity) according to Gigante et al. [34]. Of note, in this study, immunostaining performed additional to morphology on biopsy material improved the biopsy sensitivity to 48%.

As the nomenclature suggests today, cHCC-CCA shows features characteristic for both HCC and CCA and thus a tissue sample only from the HCC-like or CCA-like area might result in cHCC-CCA being misdiagnosed as HCC or CCA, respectively [11]. Multiple biopsies from different areas of tumour might prevent misclassification. However, it remains unclear how many regions should be biopsied to assure the nature of the tumour [1]. Further, according to Brunt et al., no guideline has described the minimal amount of HCC and CCA components in the biopsy specimen that is required for the diagnosis of cHCC-CCA [1]. Postoperative histopathological assessment, in which the entire resected specimen can be reviewed, still remains the gold standard method for a definite diagnosis [10]. Interestingly, as shown by Gigante et al. in a retrospective study, a two-step process which involves radiological imaging followed by a biopsy, could increase the diagnostic accuracy of cHCC-CCA, in particular when both techniques suggested the same diagnosis [34].

## 6. Histopathological Assessment 

**Figure 4:**
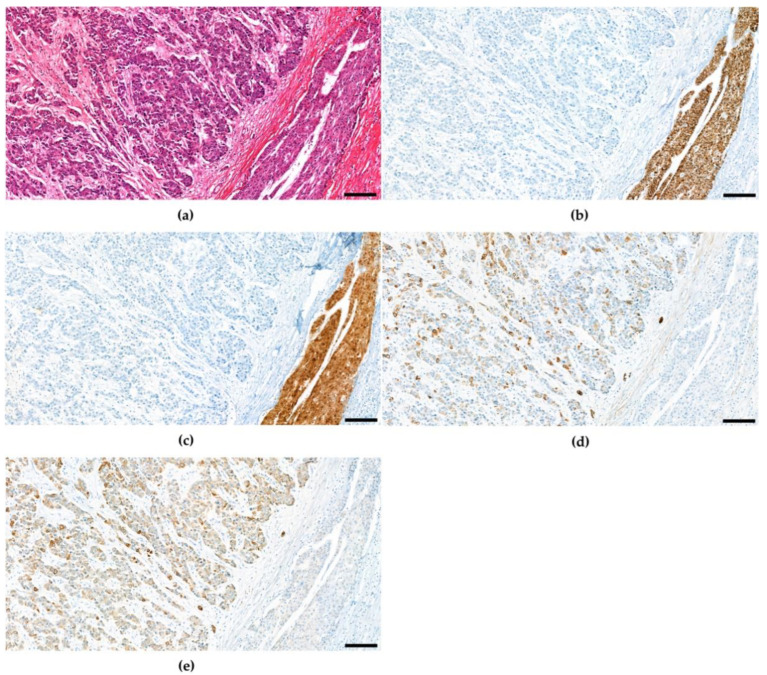
Combined hepatocellular and cholangiocellular carcinoma in a 55-year-old male patient. (**a**) H&E (**b**) HepPar1, (**c**) Arginase-1 (**d**) CK7 (**e**) CK19. CD56 and c-Kit were not expressed. Scale bar: 100 μm.

Today the diagnosis of cHCC-CCA still mainly relies on identification of unequivocal features of both hepatocytic and cholangiocytic origin based on H&E morphology, whereas hepatocytic and cholangiocytic tumour areas may show all architectural and cytological differentiation patterns described for HCC and CCA. The two components are either close to each other or deeply intermingled. Immunophenotypic expression patterns are not sufficient alone for diagnosis [46]. Immunophenotypic expression patterns may be applied to confirm cell differentiation (Figure 4). Hepatocytic and cholangiocytic differentiation may be accompanied by a variable amount of stem cells [1], which express a wide variety of unspecific immunhistochemical markers. These markers are not specific enough to make a distinct diagnosis; therefore, the classification of combined tumours with stem-cell features is no longer recommended. Nevertheless, a certain subgroup of tumours shows cells with features intermediate between HCC and CCA. These tumours are designated intermediate cell carcinomas [1,47,48]. Cholangiolocarcinomas may be a component of combined hepatocellular and cholangiocellular carcinoma; however, if cholangiolocellular carcinoma is present alone or in form of an admixture with cholangiocellular carcinoma, it is now considered subtypes of cholangiocarcinoma [49,50]. Immunohistochemical differentiation markers are commonly applied to confirm lineage differentiation. This may especially be important when available tissue is scarce [34]. Common differentiation markers include HepPar-1 [51] and Arginase-1 [52] for hepatocellular origin and CK7 and CK19 for ductular/cholangiocytic differentiation [53]. However, commonly used markers are also not entirely specific for their respective purpose. For instance, HepPar-1 may also be demonstrated in otherwise cholangiocellular differentiated areas [54]. 

## 7. Molecular Characteristics

Owing the relative rare occurrence of cHCC-CCA, little is known about molecular features of this malignancy. Despite morphological characteristics of both tumours, it long remained controversial whether cHCC-CCA genetically more closely resembles HCC or intrahepatic cholangiocarcinoma (iCCA). In 2004, Cazals-Hatem et al. conducted a genome wide allelotyping analysis of cHCC-CCA. They searched for loss of heterozygosity (LOH) in cHCC-CCA and iCCA and found that 3p and 14q LOH were common in both tumours showing that cHCC-CCA is genetically closer to iCCA than to HCC [55]. In a recent study, however, Joseph et al. performed capture-based next-generation sequencing of 20 cHCC-CCA, sequenced 10 iCCA and finally compared obtained data, demonstrating that molecular alterations characteristic for HCC were even present in iCCA components of cHCC-CCA. These finding suggested a molecular similarity of cHCC-CCA to HCC rather than CCA [56].

Nonetheless, further studies have closer investigated cHCC-CCA genomic alterations. Mutations in TP53, TERT promoter, IDH1/2 and AXIN 1, which all may also be observed in HCC and/or iCCA, were the most frequent alterations of cHCC-CCA and could be observed in 45.3–80%, 23–70%, 0–11.8% and 10% of studied cases, respectively [9,56,57]. Interestingly, in cHCC-CCA, TERT promoter mutation was consistently present in HCC and iCCA components and, therefore, is considered as an early event in the evolution of this malignancy [56]. Further, findings of the genomic and transcriptomic sequencing study by Xue et al. finally solved the previously described long-standing debate. Combined and mixed types of cHCC-CCA are different subtypes and their molecular profiles should be analysed separately. Combined type is molecularly more similar to iCCA, whereas in mixed type many HCC-like molecular features could be observed [9]. 

In addition, multiple studies provide a closer look at the frequencies of KRAS and CTNNB1 mutations in cHCC-CCA, HCC and iCCA. CTNNB1 is often mutated in HCCs (at a frequency of 6–31%) while KRAS alterations can commonly be found in iCCAs (in around 5–19%). However, both mutations occurred less frequently (CTNNB1 6–10%, KRAS 0–7.5%) in cHCC-CCA. The absence of CTNNB1 and KRAS mutations could, therefore, have diagnostic value (see Table 1) [9,56,57,58,59,60].

Continued research on molecular landscape of cHCC-CCA is warranted for a better understanding of genetic alterations patterns, as they might serve as a useful tool particularly in the diagnosis of challenging cases. Moreover, it appears reasonable to specify the role of molecular analysis in the diagnostic algorithm of cHCC-CCA. 

## 8. Future Perspectives

Despite recent advances in the molecular characterisation of cHCC-CCA, not much has changed in the last years regarding the diagnostic workup, and critical challenges remain. In the following, we have identified three areas that we think offer the greatest potential for improvements in the future.

### 8.1. Liquid Biopsies

Liquid biopsy is a new alternative approach for early diagnosis and risk stratification in patients with cancer [61]. Detection of circulating tumour-derived markers, such as circulating tumour cells (CTCs), circulating tumour DNA (ctDNA) or cell-free (cfDNA), in body fluids, is the basis of liquid biopsy and allows the identification of tumour-specific molecular alterations [62]. 

For instance, in HCC patients, it was shown that the combination of CTCs and AFP measurement had a sensitivity of 73% for identifying HCC while the AFP analysis alone had a sensitivity of 39.5% [63]. Another study reported an identical or superior accuracy of ctDNA compared to AFP in distinguishing HCC from hepatitis/cirrhosis [64]. 

In CCA patients, Shen et al. (2019) went on to compare the mutational profile obtained from the analysis of bile cfDNA to the paired tumour tissue DNA findings in 6 CCA patients. The examination of bile cfDNA revealed a high sensitivity and specificity in detecting single nucleotide variations, insertions and deletions (94.7 and 99.9%, respectively) and copy number variation (75.0 and 98.9%, respectively) suggesting bile cfDNA as a reliable source of tumour genetic landscape [65]. Thus, liquid biopsy for bile cfDNA may serve as a promising method for identifying CCA.

Importantly, a liquid biopsy has the potential to reveal the complete genomic landscape of the tumour in contrast to a tissue biopsy, which only shows a single region of the tumour with low possibility to represent all somatic mutations of the entire entity. However, to the best of our knowledge, liquid biopsies in cHCC-CCA have not been comprehensively studied.

To use liquid biopsies for cHCC-CCA, unique molecular alterations would be needed. According to Xue et al., tumour tissue of cHCC-CCAs had a much lower CTNNB1 mutation frequency (6%) compared with that of HCCs (TCGA-HCC, 27%, *p* < 0.0001). Moreover, cHCC- CCAs had a much lower KRAS mutation frequency (0%) compared with that of CCAs (ICGC-ICC, 19%, *p* < 0.0001). The scarcity of CTNNB1 and KRAS mutations was a unique feature of cHCC-CCA [9]. These observations could be a starting point for further research and a possible application in the clinical setting in case of diagnostic uncertainty. 

Overall, the analysis of molecular alterations in circulating molecules derived from tumour (e.g., CTCS, ctDNA or cfDNA) via liquid biopsy has the potential to become a distinctive non-invasive diagnostic approach that may even replace the traditional tissue biopsy, and, more importantly, detect new putative molecular mutation patterns in cHCC-CCA.

### 8.2. Radiomics

Radiomics is a rapidly developing field dealing with the extraction of quantitative radiological features in medical images. Radiomic features are, for example, tissue and lesion characteristics such as heterogeneity and shape [66,67]. Radiomics and the analysis with deep learning may prove to be a useful diagnostic tool for identifying cHCC-CCA.

Several studies applied radiomics in the analysis of liver tumour imaging [68]. In this context, for example, Yasaka et al. were able to differentiate five different classes of liver lesions using radiomics and machine learning in contrast with CT with an average accuracy of 84% [69]. Similar studies are also available for MRI [70,71]. Liu et al. found MRI enhanced with Gd-EOB or Gadovist to have an AUC of up to 0.77 (SD 0.19) for differentiating cHCC-CCA from HCC or CCA. CT was of more limited use, with an AUC of 0.64 (SD 0.17) in the delayed phase [72]. 

However, it is important to note that such models require external validation, considering variable MRI protocols and machines as well as heterogeneous patient populations. Adoption in clinical routines may also be hampered by the requirement for specialised software or, in the case of CEUS, specific examination techniques.

### 8.3. Artificial Intelligence 

Artificial intelligence (AI) describes the ability of computers to perform tasks that normally require human intelligence, such as learning or problem solving. Deep learning is a subtype of artificial intelligence, which uses models with neural networks inspired by the connections of neurons in the human brain to learn patterns in data and come up with predictions. These models are very powerful when it comes to the integration of massive amounts of heterogenous data. This data input could be, e.g., radiological imaging, patient characteristics, laboratory test results, genetic information, or histopathological images [73,74,75]. Neural networks weigh the different input data and make predictions—in this case likelihood of the presence of cHCC-CCA.

This integration of many different parameters is especially relevant in the context of cHCC-CCA since individual parameters (e.g., simultaneous elevation of AFP and CA 19-9 have a very low sensitivity for the diagnosis [24].

In HCC and CCA patients, deep learning models were applied to diverse data sets, e.g., electronic health record data, imaging modalities, histopathology and molecular biomarkers to improve the accuracy of HCC and CCA risk prediction, detection and prediction of treatment response. Numerous studies have tested the utility of deep learning for the detection of HCC/CCA, based on imaging modalities, histopathology or biomarkers. However, it should be noted that only a low number of studies integrated several heterogeneous input data in one model [76,77]. One example is the study of Zhen et al., in which the authors developed a deep learning model using clinical patient characteristics, laboratory test results and MR images from 1210 patients. The model performed well, e.g., classifying malignancies as HCC (AUC, 0.881) by just using the MR images, but the performance increased even further by integrating clinical data and laboratory tests (AUC 0.985) [78].

There are several limitations of AI models in the diagnosis of cHCC-CCA. Deep learning models are very powerful tools; however, they require a large amount of data input for the training, test and (external) validation sets [75]. Since cHCC-CCA is a rare cancer and neural networks need at least a few hundred patients (of course, depending on the specific research question), collaborations between several centres would be needed. This is especially relevant since clinical characteristics and molecular alterations of cHCC-CCA differ in various geographic regions or patient populations [11,16]. This diversity needs to be reflected in the models to obtain valid predictions. 

Although AI models have shown promising results for the detection and patient stratification in multiple cancers they are rarely used in clinical routine. To date, most of the studies used retrospective datasets, thus, more prospective studies are needed comparing their performance to existing diagnostic, staging and predictive systems [76,79].

## 9. Conclusions

cHCC-CCA is a primary liver cancer which exhibits both hepatic and biliary differentiation. Due to this nature, the diagnostic workup is particularly challenging, yet indispensable to offer optimal treatment strategies to the patients. Recent advances in the diagnosis of HCC and CCA, e.g., by using molecular information and/or artificial intelligence may provide a starting point for further cHCC-CCA studies to address current diagnostic challenges. 

## Figures and Tables

**Table 1:** Important molecular mutations and their frequencies in combined hepatocellular-cholangiocarcinoma, hepatocellular carcinoma and intrahepatic cholangiocarcinoma [9,56,57,58,59,60]. Differences in reported frequencies are likely due to cohorts from various populations. TP53, tumour protein 53; TERT, telomerase reverse transcriptase; AXIN1, axis inhibition protein 1; KMT2D, lysine methyltransferase 2D; CTNNB1, catenin beta 1; KRAS, Kirsten rat sarcoma viral oncogene homolog; IDH1, isocitrate dehydrogenase 1; IDH2 isocitrate dehydrogenase 2; FGFR2, fibroblast growth factor receptor 2; BAP1, BRCA1-associated-protein 1; ALB, albumin.

Combined Hepatocellular-Cholangiocarcinoma	Hepatocellular Carcinoma	Intrahepatic Cholangiocarcinoma
TP53 (45.3–80%) TERT promoter (23–70%) IDH1 or IDH2 (0–11.8%) AXIN1 (10%) CTNNB1 (6–10%) KMT2D (9%) KRAS (0–7.5%) FGFR2 (0–3%) BAP1 (0–3%)	TERT promoter (44–54%) TP53 (13–31%) CTNNB1 (6–31%) ALB (13–%) AXIN1 (6–8%)	IDH1 or IDH2 (9–30%) TP53 (5–22%) FGFR2 (6–20%) KRAS (5–19%) BAP1 (10–12%)
